# Is life expectancy higher in countries and territories with publicly funded health care? Global analysis of health care access and the social determinants of health

**DOI:** 10.7189/jogh.12.04091

**Published:** 2022-11-12

**Authors:** Sarah Galvani-Townsend, Isabel Martinez, Abhishek Pandey

**Affiliations:** 1 Center for Infectious Disease Modeling and Analysis (CIDMA), Yale School of Public Health, New Haven, Connecticut, USA; 2 Department of Social and Behavioral Sciences, Yale School of Public Health, New Haven, Connecticut, USA

## Abstract

**Background:**

To better understand factors influencing life expectancy, this paper examines how the availability of publicly funded health care in a country and multiple social determinants of health impact longevity of life.

**Methods:**

In this descriptive statistical analysis, data regarding publicly funded health care, life expectancy, and social determinants of health were obtained for 196 countries and 4 territories. Social determinants included 10 indicators detailing country-level information to represent 5 key categories: economic stability, education, health & health care, neighbourhood & built environment, and social & community context. Analyses consisted of: 1) comparison of mean life expectancy among countries and territories with- and without- publicly funded health care; 2) correlations in life expectancy across social determinants by health care access and level of burden; and 3) correlations in life expectancy within social determinants for health care access by level of burden.

**Results:**

Overall, life expectancy in countries and territories with- publicly funded health care (Mean (m) = 76.7 years) was significantly longer compared to countries and territories without- publicly funded health care (m = 66.8 years, *P* < 0.0001). For each social determinant, we observed longer life expectancy continued to be associated with publicly funded health care access across stratum (*P* < 0.0001), but difference in years of life expectancy existed both by burden of social determinant, as well as access to health care within quartiles of burden (Publicly funded care (yes): 68.12-80.88 years, (no): 62.39-77.33 years, all *P* < 0.05). Both social determinants as well as the availability of publicly funded health care were individually and simultaneously associated with mean longevity of life between countries and territories worldwide.

**Conclusions:**

These findings demonstrate how, if made widely available, publicly funded health care could extend longevity of life. If combined with programs to reduce the burden of social determinants, a substantial impact can be made to promote more equitable distribution of life expectancies across the world. Ultimately, both access to publicly funded care and reducing inequalities in social determinants are needed in order to promote longer and healthier aging in populations worldwide.

Major advances in the medical sciences have led to an improvement in global life expectancy, increasing from 32.0 years in 1900 to 66.3 years in 2000 [1]. Since 2000, it has continued to rise to the current 72.6 years, although stark differences exist in current life expectancies across countries and territories globally [2]. In addition, while therapies and cures have been developed to treat many life threatening conditions, individuals continue to be overburdened by treatable or preventable ailments, raising the question: what role does access to health care play in prolonging life?

While disease morbidity can play a substantial role in life expectancy, recent findings in public health research also call to attention the exigent role social determinants play for the quality and longevity of life in a population [3]. There are several individual factors intrinsically tied to a longer life expectancy around the world (eg, education, income, malnutrition) [4-8]. Such factors can interplay with access to health care in varying ways based on location, social factors, and health care system. Regressions in social determinants can also blunt the impact of medical advances and are paramount to address in order to improve the longevity and quality of life within and outside the health care setting. However, the manner in which social determinants and health care systems interact remains an underexplored area of public health.

Social determinants can be defined as modifiable factors or conditions in an individual’s daily environment that have an effect on health and which are tied to place and social context [9]. Social determinants include education, economic stability, neighbourhood and built environment, social and community context, and health care. In combination, these factors constitute five key areas of social determinants that impact the state of an individual’s health, aspects of which are all prioritized by Healthy People 2020 [10] and the Sustainable Development Goals 2030 [11]. At the forefront of these factors is education. Educational attainment impacts not only longevity of life, but also the quality of that life [12-14]. Both of these traits are central to prolonging life expectancy, as efforts are made to extend life in a manner that includes both well-being and caliber as central characteristics to a healthy aging process [15]. In the United States (US), higher educational attainment has been shown to account for as much as 30% difference in life expectancy among adults [16]. When examining life expectancy, we must also consider multiple indicators of education (eg, literacy rate, population with at least secondary education) as different factors aid us in better understanding the connection between education, health care, and life expectancy overall.

Economic stability is a second key area of social determinants that bears a substantial influence on population health. At the individual level, socioeconomic status has been shown to impact health throughout the life course [17,18]. From preterm birth [19] to healthy physical and cognitive aging [20], socioeconomic factors such as employment and income can directly and indirectly impact an individual's health. Employment status can also extend beyond the individual, seriously impacting not only their own survival, but the health and well-being of their dependents [5,14]. From a population standpoint, factors like Gross Domestic Product (GDP) are constantly tied to morbidity and mortality performance in a given population. Thus, the integration of economic stability comprises a necessary component for understanding longevity of life.

Both neighbourhood and built environment are also intrinsically tied to an individual’s life expectancy. Pollution, for example, is known to heavily impact childhood health (eg, asthma) and morbidity [21-23]. Respiratory infections, chronic diseases, and other conditions associated with air quality can also impact quality of life in youth, drastically affecting their physical health, educational attainment, and even the frequency of emergency care use. As these factors persist throughout the life course, healthy human development can be compromised causing long term effects carried into adulthood [24]. A starkly different aspect of built environment is unintentional injuries which can often lead to premature death. Road safety is one such marker which impacts rates of disability and mortality in populations around the world [25]. Globally, one person dies from a traffic-related accident every 25 seconds [26]. Unintentional injuries, combined with 6 million deaths tied to air pollution[27], make neighbourhood & built environment an integral component for understanding life expectancy.

How stressors in a person’s social and community context affect an individual can also help us to understand longevity of life. Coping strategies and other related factors give us insight into an individual’s everyday behaviour. Social indicators, such as substance abuse, can serve to highlight what proportion of a population is facing challenges in their social relationships and daily lives, and how someone copes with stressors [28]. The consumption of heavy drug and alcohol use, as seen with substance abuse, often results in adverse health effects that directly impact how long a person might live. Interrelated to substance abuse is suicide, which provides insight into how well an individual might cope with difficulties, as well as social and community challenges existing in their community. Suicide rates help to reflect the mental health of a population and access to mental health care services in that population, which contribute to and can help inform quality and longevity of life at the population level [29].

Finally, health care is an essential social determinant of health that impacts life expectancy. The state and quality of an individual’s well-being (eg, undernourishment, immunizations, health care access) has long term effects that can remain throughout the life course [30]. Research on malnutrition, for example, shows lasting effects on individual health which extend into adulthood [31]. Immunizations also play a role in life expectancy, mainly through reduction or avoidance of disease burden and transmission, thereby preventing quality of life to falter as individual’s age and in some cases reducing risk of death [32]. Through regular visits to a primary health care provider, issues like malnutrition and preventative immunizations can be identified early on, reducing the likelihood disease will develop later in life. Perhaps the most important social determinant, thus, is access to health care. Publicly funded health care can provide individuals with increased access to primary, secondary, and tertiary prevention services and treatment which can span from regular checkups to life-saving medical procedures (eg, surgeries) [33-35]. As we evaluate life expectancy, it is therefore critical to incorporate a deeper understanding of the role publicly funded health care can play for longevity of life in a population.

As noted above, these five areas of social determinants are intertwined with health and life expectancy globally. While life expectancy has been explored substantially in research among each of these areas individually, there remain two key gaps in scientific knowledge regarding the association between health care access and life expectancy. First, few studies have looked at differences that exist in health care access and its association with life expectancy in countries and territories internationally. Understanding how access to care impacts life expectancy from country-to-country is central to informing health policies around the world. Second, to our knowledge no studies have looked at the specific association between health care access, life expectancy and social determinants of health globally. Given the substantial impact social determinants of health play at the individual level, such factors like economic stability, education, neighbourhood and built environment, and social and community context may play a significant role in improving or reducing life expectancy within a country. Evaluating differences in life expectancy in countries and territories with- and without- publicly funded health care by varying levels of social determinants (eg, high literacy rate + publicly funded health care vs low literacy rate + publicly funded health care) may help us to better understand the interplay between health care systems and social determinants of health as they relate to longevity of life.

The current study sought to: 1) determine whether countries and territories with publicly funded health care have a longer mean life expectancy compared to countries and territories without publicly funded health care. We hypothesized countries and territories with access to publicly funded care will have longer mean life expectancy. 2) Evaluate whether differences exist in life expectancy by the level of social determinants among countries and territories with- and without- publicly funded health care. We expect overall life expectancy to remain longer among countries and territories with access to publicly funded care, irrespective of the level of social determinant burden they fall into, consistent with our initial hypothesis. By exploring country-level indicators for education, economic stability, neighbourhood & built environment, and social & community context in combination, we assess the association between publicly funded health care and life expectancy at different levels of social determining factors [14].

## METHODS

### Data and sources

For n = 200 countries and territories, publicly available data were collected from government and public health resources (See Table S1 in the **Online Supplement Document**) to evaluate the impact of social determinants on the association between health care access and mean population life expectancy at birth for each country. Primary data sources include the World Bank, the Central Intelligence Agency, the World Factbook, the US Census Bureau, and the United Nations (See Table S1 in the **Online Supplement Document**). For all countries and territories, data for up to 10 indicators, health care access information, as well as life expectancy were used for corresponding analyses.

### Primary variables/measures

Our primary variable of interest, health care access, was defined as the level of care provided by any given country to the general population. A country was defined to have a publicly funded health care system if it provided primary services either free of charge or at a nominal fee to all its citizens. Countries and territories were categorized by their health care systems into two groups to form a binary measure for analysis: those with publicly funded health care and those without publicly funded health care (health care access = yes/no, missing = 0).

Our primary outcome measure was life expectancy defined as the average number of years a person is expected to live given sex- and age-specific death rates prevailing at the time of that person’s birth, for a specific year in a given country [36] (life expectancy = number of years, range = 52.1-89.4 years, missing = 0).

We first assessed the association between these two measures, followed by a re-assessment that controlled for the following social determining indicators:

#### Social determinants

Social determinants included information for 10 indicators spanning education (literacy rate, secondary education), economic stability (per capita GDP, unemployment), neighbourhood & built environment (pollution, road fatalities), social & community context (male suicide rate, substance use disorders), and health care (undernourishment, infants lacking immunizations).

#### Education

Literacy rate: pertains to the proportion of individuals in a given country who are 15 years of age or older and who are able to read and write with basic comprehension (literacy rate = % of population 15+, range = 22.0%-100%, missing = 7).

Secondary education: measures all individuals in a given country who are 25 years of age or older and who have completed any secondary education (secondary education = % of population 25+ with secondary education, range = 6.1%-100%, missing = 36).

#### Economic stability

Per capita GDP: defined as the total value of goods and services produced in a country per person every year in US dollars (per capita GDP = US$ per capita, range = US$274-US$190 513 per capita, missing = 3).

Unemployment rate: represents percent of the population that is of working age and physically able to work, but not employed (unemployment rate = % of population in working age, range = 0.1%-33.4%, missing = 29).

#### Neighbourhood & built environment

Road fatalities: measures the number of deaths attributed to road collisions per 100 000 population in a country per year (road fatalities = deaths/100 000 per year, range = 0.0-35.9/100 000 per year, missing = 20).

Pollution: represented as the population-weighted annual average exposure to atmospheric particulate matter (PM) with diameters 2.5 µm (PM2.5s). Among particulate matter, those with diameters 2.5 µm are known to pose the greatest risk to an individual’s health [37] (pollution = number of PM2.5s *μg / m^3^*, range = 5.9-99.7*μg* / *m^3^,* missing = 6).

#### Social & community context

Substance use disorders: represents the proportion of a country’s population with either alcohol- or drug-use related dependence. This is measured using the International Classifications of Diseases criteria (person reporting three or more indicators of dependence for a minimum of one month within the previous year) (substance use disorders = % of persons with substance use disorders, range = 1.3%-5.9%, missing = 13).

Male suicide rate: defined as the number of deaths by suicide per 100 000 male population in a given country per year (male suicide rate = number of male deaths by suicide/100 000 per year, range = 0.0-48.3/100 000 per year, missing = 21).

#### Healthcare

Undernourishment: prevalence in the population of persons whose food intake is insufficient to meet dietary energy requirements continuously (undernourishment = % of population undernourished, range = 3.0%-62.0%, missing = 43).

Infants lacking immunizations: represents the percentage of one-year-olds in a population who lack diphtheria, tetanus, and pertussis (DTP) vaccinations (infants lacking immunization = % of infants lacking DTP immunization in population, range = 1.0%-56.0%, missing = 13).

Details on all variables data are present in Table S1 and Figures S1-S10 in the **Online Supplement Document.**

### Analyses

We first evaluated the overall association between health care access as a predictor of life expectancy across countries and territories. For Aim 1, mean life expectancy was calculated using a *t* test to compare all countries and territories with publicly funded health care access to all countries and territories without publicly funded health care access. We then evaluated our 10 social determining factors to assess if life expectancy varied by each indicator. For each indicator, we divided countries and territories into one of four groups based on quartiles of the indicator values. These groups were then ranked from 1-4 based on how well countries and territories within each group were performing for that value. For example, for literacy rate which ranges from 22.0%-100%, quartile 1 includes countries and territories with 0%-25% literacy. For this indicator, quartile 1 is also rank 1 while quartile 4 (75%>literacy) is rank 4. For unemployment, however, rank 1 corresponds to quartile 4 (75%-100% unemployment) indicating the highest burden of unemployment (see Table S2 in the **Online Supplement Document**). These ranks were used to evaluate countries and territories’ performance in analyses. For Aim 2, we ran one-way multiple analysis of variance (MANOVA) to assess social determinants as independent predictors of life expectancy across ranks. Next, we used two-way *t* tests to evaluate differences in life expectancy within ranks for each social determining indicator. Finally, we evaluated both health care access and social determining indicators as predictors of life expectancy using two-way MANOVA models. For all tests conducted, we set the level of significance to be 0.05. Analyses were conducted using SAS 9.4 software and Python 3.5.

## RESULTS

**Figure 1:**
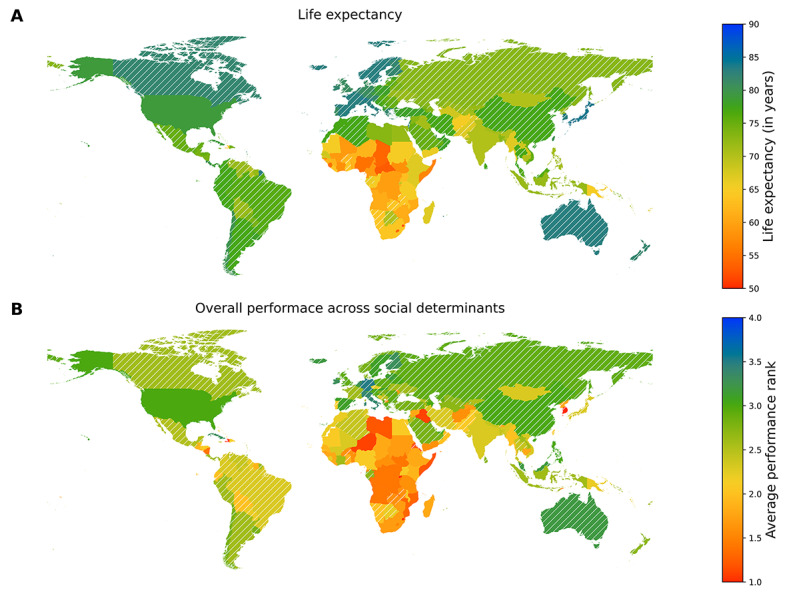
**Panel A.** Life expectancy. **Panel B.** Overall performance of social determinants across the world. Hatched lines represent the countries and territories with publicly funded health care systems. Overall performance across social determinants of a country is calculated as the average of that country’s performance ranks across the 10 social determining indicators considered in the analysis.

Overall, we found a substantial variation in life expectancy around the world ranging from 51.2 years in Eswatini to 89.4 years in Monaco (see **Figure 1**, panel A). Of 196 countries and 4 major territories evaluated, 60.5% (n = 121) had some form of publicly funded health care. Life expectancy was >80 years in 18.5% (n = 37) of countries and territories, and <60 years in 7% (n = 14) of countries and territories. For Aim 1, we found mean life expectancy among countries and territories with publicly funded health care was significantly higher (m = 76.7 years, standard deviation (SD) = 5.3, range (r) = 62.0-89.4 years) compared to countries and territories without publicly funded health care (m = 66.8 years, SD = 6.7, r = 52.1-82.0 years, *P* < 0.05) confirming our initial hypothesis.

**Figure 1**, panel B, displays the summation of ranks of social determining factors for each country. Similar to life expectancy, overall performance of countries and territories for various social determining factors also varies globally. Visually, certain geographical areas with lower life expectancy (eg, Sub-saharan Africa = 51.2-55.0 years) overlapped with areas showing poorer overall performance of social determining factors (performance rank = 0-1, example: lowest quartile of per capita GDP, range = US$274-US$1905.30 per year). As such, we explored the impact of social determining factors further by country’s performance using one-way MANOVAs (Aim 2, **Table 1**). For literacy rate (f = 20.36), population with secondary education (f = 45.60), per capita GDP (f = 88.16), road fatalities (f = 55.77), substance use disorders (f = 9.08), and undernourishment (f = 57.32,), the overall life expectancy across ranks was significantly different (*P* < 0.05). For unemployment rate (f = 2.29, *P* = 0.08), pollution (f = 2.39, *P* = 0.07), male suicide rates (f = 1.94, *P* = 0.13) and infants lacking immunizations (F = 1.58, *P* = 0.19), differences in life expectancy between ranks were not significant.

**Table 1:** One way and two-way Manova

MANOVA	a) One way	b) Two-way		
Social indicator (single predictor)	Social indicators (F, P)	Healthcare access	Overall (F, P)
Literacy rate	20.36, *P* < 0.05	9.20, *P* < 0.05	90.00, *P* < 0.05	44.96, *P* < 0.05
Population with Secondary education	45.60, *P* < 0.05	17.17, *P* < 0.05	30.80, *P* < 0.05	48.27, *P* < 0.05
GDP per capita	88.16, *P* < 0.05	41.82, *P* < 0.05	29.35, *P* < 0.05	83.17, *P* < 0.05
Unemployment	2.29, ***P* = 0.08**	2.01, ***P* = 0.11**	113.70, *P* < 0.05	31.30, *P* < 0.05
Road fatalities	55.77, *P* < 0.05	22.40, *P* < 0.05	29.55, *P* < 0.05	56.00, *P* < 0.05
Pollution	2.39, ***P* = 0.07**	0.81, ***P* = 0.49**	108.60, *P* < 0.05	30.02, *P* < 0.05
Substance use	9.08, *P* < 0.05	5.08, *P* < 0.05	104.61, *P* < 0.05	36.81, *P* < 0.05
Male suicide rate	1.94, ***P* = 0.13**	00.73, ***P* = 0.54**	99.41, *P* < 0.05	27.12, *P* < 0.05
Undernourishment	57.32, *P* < 0.05	25.41, *P* < 0.05	21.73, *P* < 0.05	54.25, *P* < 0.05
Infants lacking immunizations	1.58, ***P* = 0.19**	0.78, ***P* = 0.50**	109.60, *P* < 0.05	29.29, *P* < 0.05

To evaluate differences within ranks by health care access (Aim 2), we ran two-way *t* tests to compare within- group means for life expectancy (**Table 2**). countries and territories with publicly funded health care had significantly higher mean life expectancy compared to countries and territories without publicly funded health care. This was irrespective of performance rank for all social determining factors with the exceptions of per capita GDP (1 significant group) and undernourishment (2 significant groups). For per capita GDP, countries and territories with the lowest performance (rank 1, US$274-US$1905.30) saw a difference of 7.04 years in life expectancy (*P* < 0.05). Similarly, undernourishment in rank 1 had a difference of 4.60 years (*P* < 0.05) and in rank 2 a difference of 6.21 years (*P* < 0.05) for life expectancy between groups. Countries and territories with higher per capita GDP (rank 2-4) and low (3%-6% of population) burden of undernourished population (rank 3-4) had no significant difference by health care access for life expectancy, although mean life expectancy remained higher in countries and territories with publicly funded health care.

**Table 2:** Two-way *t* test across performance ranks for each social determining factor

	Performance rank									Performance rank							
	**1**		**2**		**3**		**4**			**1**		**2**		**3**		**4**	
*Healthcare access*	*Yes*	*No*	*Yes*	*No*	*Yes*	*No*	*Yes*	*No*		*Yes*	*No*	*Yes*	*No*	*Yes*	*No*	*Yes*	*No*
**Education**									**Social & community context**								
**Literacy rate** *m (SD)*	72.84 (6.50)	63.90 (5.86)	76.17 (5.35)	68.16 (6.75)	78.08 (4.30)	70.12 (7.20)	77.67 (4.47)	63.07 (6.26)	**Substance use disorders**	77.35 (3.92)	73.27 (4.56)	75.64 (5.12)	68.92 (7.39)	75.95 (5.60)	62.39 (4.57)	74.53 (5.78)	67.26 (6.43)
*t, P value*	*t* = -4.83 *P* < 0.05		*t* = -4.90 *P* < 0.05		*t* = -3.82 *P* < 0.05		*t* = -3.91 *P* < 0.05			*t* = -2.84 *P* < 0.05		*t* = -3.35 *P* < 0.05		*t* = -8.94 *P* < 0.05		*t* = -3.54 *P* < 0.05	
**Secondary education**	70.83 (6.11)	63.91 (5.87)	74.72 (5.23)	69.91 (5.52)	78.53 (4.12)	72.71 (5.53)	77.49 (4.45)	72.50 (4.64)	**Male suicide**	76.00 (6.01)	67.41 (6.13)	77.24 (4.57)	62.87 (6.94)	76.00 (5.99)	67.38 (6.15)	76.17 (5.06)	71.55 (6.19)
	*t* = -2.65 *P* < 0.05		*t* = -2.81 *P* < 0.05		*t* = -3.21 *P* < 0.05		*t* = -2.52 *P* < 0.05			*t* = -4.64 *P* < 0.05		*t* = -8.44 *P* < 0.05		*t* = -4.74 *P* < 0.05		*t* = -2.49 *P* < 0.05	
**Economic stability**									**Health & health care**								
**GDP per capita**	70.37 (6.65)	63.33 (5.15)	73.14 (4.59)	70.06 (5.74)	75.38 (3.29)	70.33 (6.28)	80.88 (3.39)	77.33 (5.69)	**Undernourishment**	68.12 (3.60)	63.52 (5.83)	74.69 (5.87)	68.48 (5.83)	75.07 (3.45)	71.00 (6.52)	78.86 (3.94)	77.25 (4.03)
	*t* = -3.75 *P* < 0.05		*t* = -2.00 *P* = 0.05		*t* = -1.93 *P* = 0.11		*t* = -1.69 *P* = 0.10			*t* = -2.11 *P* < 0.05		*t* = -3.20 *P* < 0.05		*t* = -1.73 *P* = 0.11		*t* = -0.79 *P* = 0.43	
**Unemployment**	76.49 (5.06)	65.94 (7.59)	72.27 (5.18)	67.50 (5.90)	77.61 (5.09)	70.44 (6.66)	76.61 (5.69)	64.76 (6.86)	**Infants lacking immunizations**	76.62 (5.00)	65.07 (7.04)	75.11 (6.51)	67.82 (8.14)	78.15 (5.28)	65.46 (6.69)	76.35 (4.25)	69.11 (4.78)
	*t* = -5.37 *P* = <0.05		*t* = -5.76 *P* < 0.05		*t* = -3.95 *P* < 0.05		*t* = -6.17 *P* < 0.05			*t* = -6.36 *P* < 0.05		*t* = -3.37 *P* < 0.05		*t* = -6.41 *P* < 0.05		*t* = -5.82 *P* < 0.05	
Neighbourhood & built environment									*m = mean life expectancy (years)*								
**Pollution**	75.07 (5.88)	65.94 (6.30)	76.44 (5.69)	67.89 (6.64)	76.98 (4.57)	65.59 (9.72)	77.45 (5.51)	66.79 (6.65)	SD = standard deviation of mean								
	*t* = -5.08 *P* < 0.05		*t* = 4.65 *P* < 0.05		*t* = -3.91 *P* = 0.05		*t* = -5.67 *P* < 0.05										
**Road fatalities**	70.75 (6.76)	63.59 (5.69)	73.48 (4.24)	68.46 (7.20)	76.00 (3.51)	73.22 (3.23)	80.06 (4.62)	71.67 (9.07)									
	*t* = -3.11 *P* < 0.05		*t* = -2.92 *P* < 0.05		*t* = -2.16 *P* < 0.05		*t* = -2.86 *P* < 0.05										

M – mean life expectancy (years), SD – standard deviation of mean

To evaluate health care access and social determining indicator performance ranks for their effect on life expectancy (Aim 2), we used two-way MANOVA models. Each MANOVA included two independent variables (social determining factor performance, health care access) and life expectancy as the dependent variable. Across all models, health care access was a significant predictor of life expectancy after accounting for social determining indicators (**Table 1**). For social determinants, literacy rate, population with secondary education, per capita GDP, road fatalities, substance use disorders, and undernourishment were all significant predictors of life expectancy after accounting for the effect of health care access (*P* 0.05 for all). Similar to the one-way MANOVA models, for unemployment (*P* = 0.11), pollution (*P* = 0.49) male suicide rate (*P* = 0.53) and infants lacking immunizations (*P* = 0.50), two-way MANOVA models were not significant.

## DISCUSSION

Overall, we found mean life expectancy to be higher in countries and territories with publicly funded health care compared to those without, confirming our initial hypothesis. This trend persisted across social determinants with some notable findings. From our benchmark assessment, there was an apparent overlap in areas with shorter life expectancy and areas with poor performance of social determinants. Upon statistical evaluation, it became clear that regardless of the specific stratum of social determinant or corresponding performance rank, countries and territories with publicly funded health care access continued to have longer life expectancy. For example, in looking at GDP and health care access, among countries and territories with some form of publicly funded health care, life expectancy in the highest performance rank (rank 4) was 10.5 years longer than life expectancy for countries and territories in the lowest performance rank (rank 1). When comparing countries and territories without publicly funded health care access, the difference in life expectancy was even greater (14.0 years, rank 1 vs rank 4). Even more alarming, when looking at the most distinct stratum, countries and territories in rank 1 without health care access compared to countries and territories in rank 4 with health care access had a difference in life expectancy of 17.55 years. This highlights the interplay of health care access with factors impacting quality of life, both of which uniquely affect longevity of life.

When considering how health care access affects longevity of life, social determinants provide insight into what adversity an environment presents for health. At the individual level, a person's ability to navigate health care systems is directly impacted by the socioeconomic status of their country. If we look at the GDP for ranks 2-4, life expectancy did not vary significantly by access to publicly funded health care implying good economic conditions in a country can mitigate some of the challenges associated with access to health care at the individual level. How easily a person is able to, for example, obtain a hospital bed and the quality of care delivered will vary drastically based on a country's level of economic prosperity. A country with a stronger per capita GDP may benefit from improved access to health technology and infrastructure, ensuring improved preventative care is obtained as compared to resource-low settings. At high levels of GDP there will be less of a burden from social determinants affecting the health of a population. Conversely, if a country has much lower GDP and fares poorly from a socioeconomic standpoint, the role that health care access plays may carry more importance because of the adversity presented by social determinants in this context. As such, while health care access remains the most important factor, we cannot ignore the substantial impact social determinants can play in promoting or hindering quality of life and health.

### Access to health care & improving social conditions – a two-sided approach

Publicly funded health care is a determinant of health. It reduces individual cost of health care, while enabling more equitable access. Not having publicly funded health care can actually create barriers to care, particularly for low income and other vulnerable populations. Lack of publicly funded health care often delays diagnosis of diseases, which has the potential to turn chronic conditions into fatal outcomes. As seen in the HIV care continuum, aggressive cancers, and even the current COVID-19 pandemic, earlier entry into care can be pivotal for lowering disability, morbidity, and mortality risk.

Reluctance of vulnerable groups often stems from an inability to take on the financial burdens or responsibilities of obtaining care, leaving their health to be subjected to using high-cost emergency services when conditions have escalated in both severity and consequently cost. From a health policy standpoint, the decision between small costs that have to be exchanged for present needs for individuals or families, could be curtailed by government funded programs that prevent expensive emergency situations and avoidable disabilities or deaths from occurring. If we consider the “Ayushman Bharat” program in India, a lower-middle-income country, there are notable changes taking place. The program provides financial assistance for health care services to families in need and covers almost all secondary hospitalizations, as well as many tertiary [38]. The plan covers almost 40% of the population, ensuring that everyone gets the medical attention that they need. India has also set up 150 000 wellness centres that provide services including pregnancy care and maternal health services, neonatal and infant health services, and child health services. As a result, eligible individuals are able to present themselves to health care facilities on their own accord. Over 10 million people have already benefited from this initiative since it began in September 2018. Programs like this could be developed in any country with poor or disparate access to health care. For example, in low-income parts of Turkey, everyone is required to pay for their health-related expenditure, which results in people often delaying visits to health care facilities [39]. The same is the case in the US, the only high-income country without publicly funded health care, where people lacking insurance (eg, immigrants) also often delay entry into care [40-42]. For both of these settings, even partially covered health care could increase access for millions of individuals for an array of social determining outcomes and conditions.

In our analysis of 194 countries and 4 major territories, we found a difference between quartiles for longevity of life across all 10 measures. The greatest divides can be seen in countries and territories with high levels of pollution (>75%), low to moderate drug use (25%-50%), and countries and territories with a high proportion of infants lacking immunizations (>75%). For these variables, these data might be regarded as key correlates of health. Our results suggest that publicly funded health care does, in fact, impact life expectancy and that social determinants can play a significant role in improving or reducing the mean number of years of life expectancy in a given population. While not all social determinants play the same role for improving health (eg, male suicide rate, infants lacking immunizations), variations seen across ranks demonstrate that all social determinants remain important factors affecting life. As such, access to free or universal health care will in fact universally improve life expectancy if made widely accessible around the world and – wherever needed – should be accompanied by efforts to reduce the burden of social determinants of health placed on the health of a population.

### Life expectancy and implications of the COVID-19 pandemic

In the last two years, COVID-19 and its variants have killed over six million people worldwide. As a result, it has also impacted life expectancy, including a drop of 1.86 years in the US, which is largely driven by racial disparities occurring in Black and Latino populations (eg, Black: -2.10 years, Latino: -3.05 years) [43]. Similarly in Brazil, the female-to-male gap in life expectancy has widened by 9.1% due to gender-based differences in COVID-19 risk. The pandemic has also led to a greater decline in life expectancy in regions disparately impacted by social determinants, such as income inequality, lack of access to infrastructure, and shortage of physicians and hospital beds [44]. For some rural areas in Brazil, progress in life expectancy has seen as much as a 60% reduction during the pandemic, progress that took nearly 20 years to achieve. This serves to highlight the vulnerability of subpopulations particularly afflicted by social determining factors (eg, racial/ethnic disparities in deaths) [45], underscoring the existing weaknesses in health care systems today.

As we consider the COVID-19 pandemic and uncertainty towards vaccinations, parallels for the potential impact of uptake of immunizations is highlighted in our findings for infants lacking immunizations. When evaluating performance of infants lacking immunizations among countries and territories across ranks, there does not appear to be significant differences in life expectancy as with other social determinants. For countries and territories with publicly funded health care, the difference in longevity of life in rank 1 compared to rank 4 is negligible, and the difference among countries and territories without publicly funded care is only 4.04 years. However, when looking at differences within the lowest performance rank (rank 1) by access to publicly funded health care we see a stark difference of 11.55 years of life. These larger differences persisted across ranks 2, 3 and 4, underscoring the unequivocal role a health care system can play for life expectancy. Despite having the highest annual health care expenditure globally, the US fails to achieve a life expectancy comparable to countries and territories such as England or Costa Rica, both of which have publicly funded health care systems [46-48]. Addressing disparities in infant immunizations could contribute to improving life expectancy through reduced childhood morbidity and mortality rates, ultimately improving life expectancy in the US and countries and territories alike.

### Study limitations

While there is much to be learned through this global evaluation of life expectancy, this study is not without its limitations. First, for several social determining factors, publicly available data was not accessible for every country. Due to data restrictions, we were unable to obtain variable information for each country from the same year. To address this, we utilized the latest available information for all metrics. We also mitigated data limitations by including missing data in our statistical analyses, which served to underestimate rather than overestimate any effects. Additionally, data quality and control at the country level may also impact some correlations drawn in this study. However, inaccuracies contained in the data may be alleviated by our use of quartiles which eliminates the reliance on individual country data. Similarly, we were only able to incorporate a binary measure for health care access into our analysis despite global variations between health care systems. Amongst countries and territories with publicly funded health care systems, delivery, access and costs associated with health care vary. These heterogeneities in the quality of health care delivery to individuals must be further investigated to fully understand the impact health care access has on life expectancy. Although we used elementary classifications, our findings still highlight just how important any form of publicly funded health care access is to a populations’ health.

Given the multitude of social determinants that may influence health, we were unable to include an exhaustive list of factors. Factors included in the study were driven by known correlations with life expectancy and data availability. GDP per capita, one of our socioeconomic factors, is an example of a readily available and standard measure to compare between countries. While it does not account for the distribution of a country’s wealth, when analysed alongside the unemployment rate, these capture biases at either end of the economic spectrum (eg, the 1%, poverty). Thus, our use of multiple indicators allows us to mitigate biases that would be otherwise introduced by the use of a single indicator.

Although our analyses had these limitations, we were able to incorporate multiple indicators for 5 key millennium development goal areas. We were also able to evaluate the simultaneous impact of indicators and health care access on life expectancy. These associations are all influenced by specific cultural, community, and socioeconomic environments, relationships that we only begin to address in our assessment. This study highlights the critical urgency to focus public health research and efforts on understanding the heterogeneity of populations and health and what role health care access – in all its iterations – has for quality and longevity of life. To our knowledge, this study is the first to evaluate globally the relationship between health care access, social determinants, and life expectancy. Future research should continue to explore analytic approaches that may be better able to capture such associations.

## CONCLUSIONS

As public health and medicine continue to develop new advancements to promote health and longevity of life, we must also continue to ensure inclusivity in access to those advancements and quality of life globally. These findings highlight how substantial an impact even basic access to publicly funded care can make in improving life expectancy despite substantial social and economic burdens. Our findings also demonstrate just how pivotal social determinants can be for life expectancy. If we are to make significant headway in extending life, we must consider a dual-prong approach that includes both access to health care and reduced burden of social determinants to synergistically improve health throughout the life course. Only by incorporating both equity of social determinants and access to health care can we provide populations with the best possible outcomes for their health and longevity of life.

## Additional material


Online Supplementary Document

## References

[1] Roser M, Ortiz-Ospina E, Ritchie H. Life Expectancy. Our World in Data. 2013. Available: https://ourworldindata.org/life-expectancy. Accessed: 26 January 2022.

[2] WHO. | Life expectancy. 2018. Available: http://www.who.int/gho/mortality_burden_disease/life_tables/situation_trends_text/en/. Accessed: 27 December 2019.

[3] Braveman P Gottlieb L The social determinants of health: it’s time to consider the causes of the causes. Public Health Rep. 2014;129:19. 10.1177/00333549141291S206 24385661 PMC3863696

[4] Chetty R Stepner M Abraham S Lin S Scuderi B Turner N The Association Between Income and Life Expectancy in the United States, 2001-2014. JAMA. 2016;315:1750-66. 10.1001/jama.2016.4226 27063997 PMC4866586

[5] Assari S Life Expectancy Gain Due to Employment Status Depends on Race, Gender, Education, and Their Intersections. J Racial Ethn Health Disparities. 2018;5:375-86. 10.1007/s40615-017-0381-x 28634876 PMC6392452

[6] Uchendu FN Hunger influenced life expectancy in war-torn Sub-Saharan African countries. J Health Popul Nutr. 2018;37:11. 10.1186/s41043-018-0143-3 29703244 PMC5921790

[7] India State-Level Disease Burden Initiative Malnutrition Collaborators The burden of child and maternal malnutrition and trends in its indicators in the states of India: the Global Burden of Disease Study 1990-2017. Lancet Child Adolesc Health. 2019;3:855-70. 10.1016/S2352-4642(19)30273-1 31542357 PMC6839043

[8] Hummer RA Hernandeu EM The Effect of Educational Attainment on Adult Mortality in the United States*. Popul Bull. 2013;68:1. 25995521 PMC4435622

[9] Centers for Disease Control and Prevention (CDC). | Social Determinants of Health: Know What Effects Health. 2021. Available: https://www.cdc.gov/socialdeterminants/index.htm. Accessed: 2 March 2022.

[10] Healthy People. 2020|Social Determinants of Health.2020. Available: https://www.healthypeople.gov/2020/topics-objectives/topic/social-determinants-of-health. Accessed: 27 Dec 2019.

[11] United Nations. | The 17 goals. 2022. Available: https://sdgs.un.org/goals. Accessed: 11 Feb 2022.

[12] Hoque MM King EM Montenegro CE Orazem PF Revisiting the relationship between longevity and lifetime education: global evidence from 919 surveys. J Popul Econ. 2019;32:551-89. 10.1007/s00148-018-0717-9

[13] Clouston S Richards M Smith D Mukherjee S Zhang Y Hou W Education and the onset of cognitive pathology: a longitudinal analysis of accelerated cognitive decline. Innov Aging. 2018;174:47-47. 10.1093/geroni/igy023.174

[14] Kabir M Determinants of Life Expectancy in Developing Countries. J Dev Areas. 2008;41:185-204. 10.1353/jda.2008.0013

[15] Giacalone D Wendin K Kremer S Frøst MB Bredie WLP Olsson V Health and quality of life in an aging population – Food and beyond. Food Qual Prefer. 2016;47:166-70. 10.1016/j.foodqual.2014.12.002

[16] Meara ER Richards S Cutler DM The gap gets bigger: changes in mortality and life expectancy, by education, 1981-2000. Health Aff (Millwood). 2008;27:350-60. 10.1377/hlthaff.27.2.350 18332489 PMC2366041

[17] Frytak JR, Harley CR, Finch MD. Socioeconomic Status and Health over the Life Course. Handbooks of Sociology and Social Research; 2003.

[18] Smith JP The Impact of Socioeconomic Status on Health over the Life-Course. J Hum Resour. 2007;XLII:739-64. 10.3368/jhr.XLII.4.739

[19] Socioeconomic Disparities in Adverse Birth Outcomes A Systematic Review. Am J Prev Med. 2010;39:263-72. 20709259 10.1016/j.amepre.2010.05.012

[20] Hurst L Stafford M Cooper R Hardy R Richards M Kuh D Lifetime socioeconomic inequalities in physical and cognitive aging. Am J Public Health. 2013;103:1641-8. 10.2105/AJPH.2013.301240 23865666 PMC3780680

[21] Hansel NN Romero KM Pollard SL Bose S Psoter KJ Underhill L Ambient Air Pollution Adversely Impacts Various Domains of Asthma Morbidity among Peruvian Children. Ann Am Thorac Soc. 2019;16:348-55. 30365919 10.1513/AnnalsATS.201807-448OC PMC6394121

[22] Psoter KJ De Roos AJ Wakefield J Mayer JD Rosenfeld M Air pollution exposure is associated with MRSA acquisition in young U.S. children with cystic fibrosis. BMC Pulm Med. 2017;17:106. 10.1186/s12890-017-0449-8 28750627 PMC5530959

[23] Samoli E Dimakopoulou K Evangelopoulos D Rodopoulou S Karakatsani A Veneti L Is daily exposure to ozone associated with respiratory morbidity and lung function in a representative sample of schoolchildren? Results from a panel study in Greece. J Expo Sci Environ Epidemiol. 2017;27:346-51. 10.1038/jes.2016.32 27189255

[24] Cummins SK Jackson RJ THE BUILT ENVIRONMENT AND CHILDREN’S HEALTH. Pediatr Clin North Am. 2001;48:1241-52. 10.1016/S0031-3955(05)70372-2 11579672

[25] Dhondt S Pirdavani A Macharis C Bellemans T Putman K Translating road safety into health outcomes using a quantitative impact assessment model. Inj Prev. 2012;18:413-20. 10.1136/injuryprev-2011-040286 22729161

[26] Chandran A Kahn G Sousa T Pechansky F Bishai DM Hyder AA Impact of road traffic deaths on expected years of life lost and reduction in life expectancy in Brazil. Demography. 2013;50:229-36. 10.1007/s13524-012-0135-7 23011943

[27] Environmental Defense Fund|Health impacts of air pollution. Available: https://www.edf.org/health/health-impacts-air-pollution. Accessed: 11 Febuary 2022.

[28] Canino G Vega WA Sribney WM Warner LA Alegría M Social Relationships, Social Assimilation, and Substance-Use Disorders among Adult Latinos in the U.S. J Drug Issues. 2008;38:69-101. 10.1177/002204260803800104 20011228 PMC2792759

[29] Hedegaard H Curtin SC Warner M Suicide Mortality in the United States, 1999-2017. NCHS Data Brief. 2018;(330):1-8. 30500324

[30] Diener E Chan M Happy People Live Longer: Subjective Well-Being Contributes to Health and Longevity. Appl Psychol Health Well-Being. 2011;3:1-43. 10.1111/j.1758-0854.2010.01045.x

[31] WHO. | Road traffic injuries. 2019. Available: https://www.who.int/news-room/fact-sheets/detail/road-traffic-injuries. Accessed: 1 December 2019.

[32] Andre FE Booy R Bock HL Clemens J Datta SK John TJ Vaccination greatly reduces disease, disability, death and inequity worldwide. Bull World Health Organ. 2008;86:140-6. 10.2471/BLT.07.040089 18297169 PMC2647387

[33] Assi R Özger-İlhan S İlhan MN Health needs and access to health care: the case of Syrian refugees in Turkey. Public Health. 2019;172:146-52. 10.1016/j.puhe.2019.05.004 31235210

[34] Hao L Xu X Dupre ME Guo A Zhang X Qiu L Adequate access to healthcare and added life expectancy among older adults in China. BMC Geriatr. 2020;20:129. 10.1186/s12877-020-01524-9 32272883 PMC7146971

[35] Burns FM Johnson AM Nazroo J Ainsworth J Anderson J Fakoya A Missed opportunities for earlier HIV diagnosis within primary and secondary healthcare settings in the UK. AIDS. 2008;22:115-22. 10.1097/QAD.0b013e3282f1d4b6 18090399

[36] WHO. |Life expectancy at birth (years).2021. Available: https://www.who.int/data/gho/indicator-metadata-registry/imr-details/65. Accessed: 16 November 2021.

[37] Epa US. OAR. Particulate matter (PM) basics. 2016. Available: https://www.epa.gov/pm-pollution/particulate-matter-pm-basics. Accessed: 15 November 2021.

[38] Lahariya C “Ayushman Bharat” Program and Universal Health Coverage in India. Indian Pediatr. 2018;55:495-506. 10.1007/s13312-018-1341-1 29978817

[39] Kisa A Yilmaz F Younis MZ Kavuncubasi S Ersoy K Rivers PA Delayed use of healthcare services among the urban poor in Turkey. Public Health Rep. 2009;39:88. 10.1108/17537980910981796

[40] Horton S Cole S Medical returns: seeking health care in Mexico. Soc Sci Med. 2011;72:1846-52. 10.1016/j.socscimed.2011.03.035 21531062

[41] Derose KP Escarce JJ Lurie N Immigrants and health care: sources of vulnerability. Health Aff (Millwood). 2007;26:1258-68. 10.1377/hlthaff.26.5.1258 17848435

[42] Ayón C Ramos Santiago J López Torres AS Latinx Undocumented Older Adults, Health Needs and Access to Healthcare. J Immigr Minor Health. 2020;22:996-1009. 10.1007/s10903-019-00966-7 31898077

[43] Andrasfay T Goldman N. Reductions in 2020 US life expectancy due to COVID-19 and the disproportionate impact on the Black and Latino populations. Proc Natl Acad Sci U S A. 2021;118:e2014746118. 10.1073/pnas.2014746118 33446511 PMC7865122

[44] Castro MC Gurzenda S Turra CM Kim S Andrasfay T Goldman N Reduction in life expectancy in Brazil after COVID-19. Nat Med. 2021;27:1629-35. 10.1038/s41591-021-01437-z 34188224 PMC8446334

[45] Parpia AS Martinez I El-Sayed AM Wells CR Myers L Duncan J Racial disparities in COVID-19 mortality across Michigan, United States. EClinicalMedicine. 2021;33:100761. 10.1016/j.eclinm.2021.100761 33718849 PMC7933264

[46] Rozée EMA. National life tables – life expectancy in the UK - Office for National Statistics. Office for National Statistics. 23 Sep 2020. Available: https://www.ons.gov.uk/peoplepopulationandcommunity/birthsdeathsandmarriages/lifeexpectancies/bulletins/nationallifetablesunitedkingdom/2017to2019. Accessed: 23 June 2021.

[47] Rosero-Bixby L The exceptionally high life expectancy of Costa Rican nonagenarians. Demography. 2008;45:673-91. 10.1353/dem.0.0011 18939667 PMC2831395

[48] Rosero-Bixby L Dow WH Exploring why Costa Rica outperforms the United States in life expectancy: A tale of two inequality gradients. Proc Natl Acad Sci U S A. 2016;113:1130-7. 10.1073/pnas.1521917112 26729886 PMC4747769

