# Supplementary material for: Is life expectancy higher in countries and territories with publicly funded health care? Global analysis of health care access and the social determinants of health
Source: J Glob Health. 2022 Nov 12;12:04091. doi: 10.7189/jogh.12.04091 (PMC9653205; doi:10.7189/jogh.12.04091)
Supplement: Online Supplementary Document [file jogh-12-04091-s001.pdf]

ONLINE SUPPLEMENTARY DOCUMENT

Title: Is life expectancy higher in countries and territories with publicly funded healthcare?: Global analysis of healthcare access and the social determinants of health

Authors: Sarah Galvani-Townsend, Isabel Martinez, Abhishek Pandey

Table S1. Social determinant variables, definitions, and data source.

Category	Metric	Definition	Source
**Education**	Literacy Rate	Adult literacy rate is the percentage of people ages 15 and above who can read and write with understanding a short simple statement about their everyday life	1,2
	Population Secondary Education	Percentage of population ages 25 and older with at least some secondary education	3,4
**Economic stability**	GDP Per Capita	The total value of goods and services produced in a country per person in USD every year.	5
	Unemployment	Proportion of individuals seeking employment in the labor force (people of working age)	6,7
**Neighborhood & built environment**	Pollution	National-level exposures to PM2.5: Population-weighted annual averages in µg/m3 unites	8,9
	Road Fatalities	Road fatalities per 100,000 inhabitants per year	10,11
**Social & community context**	Drug Use	Proportion of the population with alcohol or drug use related disorders	12
	Male Suicide Rate	Suicide rate per 100,000 male population	13,14
**Health & Healthcare**	Undernourishment	Prevalence of undernourishment as a percentage of population	15,16
	Infants Lacking Immunization	Percentage of one-year-olds lacking DTP immunization	17

More detailed information including raw data, year and reference is present at https://doi.org/10.5281/zenodo.7057779

Table S2: Performance Ranking

Category	Metric	Quartiles			
		<25%	25–50%	50–75%	>75%
**Education**	Literacy Rate	1	2	3	4
	Population Secondary Education	1	2	3	4
**Economic stability**	GDP Per Capita	1	2	3	4
	Unemployment	4	3	2	1
**Neighborhood & built environment**	Pollution	4	3	2	1
	Road Fatalities	4	3	2	1
**Social & community context**	Drug Use	4	3	2	1
	Suicide Rate Male	4	3	2	1
**Health & Healthcare**	Undernourishment	4	3	2	1
	Infants Lacking Immunization	4	3	2	1

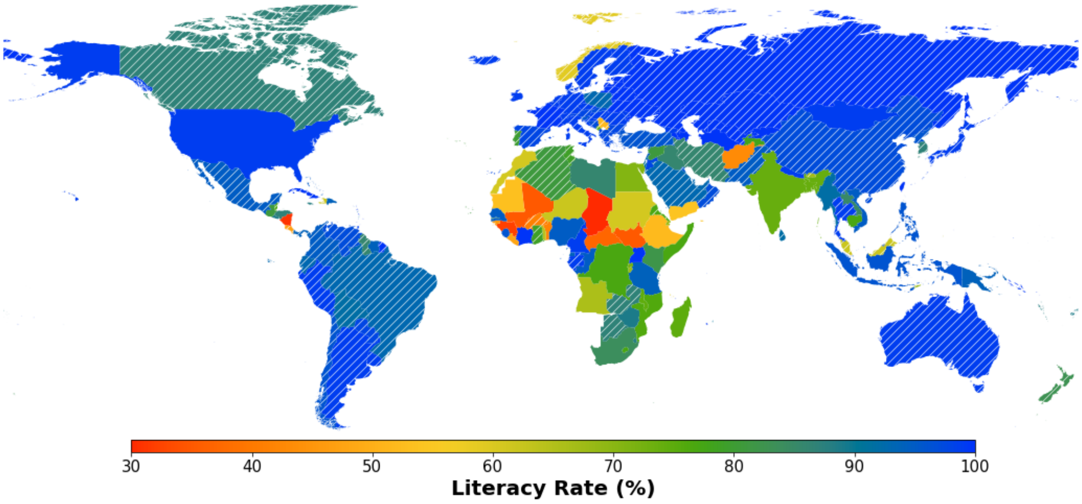

Figure S1. The country-specific percentage of people ages 15 and above who can read and write with understanding a short simple statement about their everyday life. The hatched lines represent countries with publicly funded healthcare. Countries without data are represented in gray.

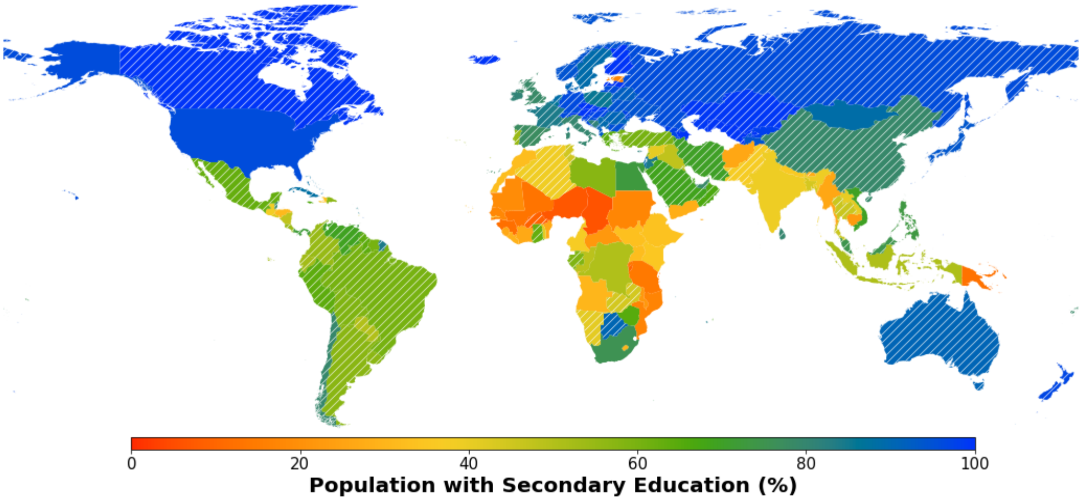

Figure S2. The country-specific percentage of population ages 25 and older with at least some secondary education. The hatched lines represent countries with universal healthcare. Countries without data are represented in gray.

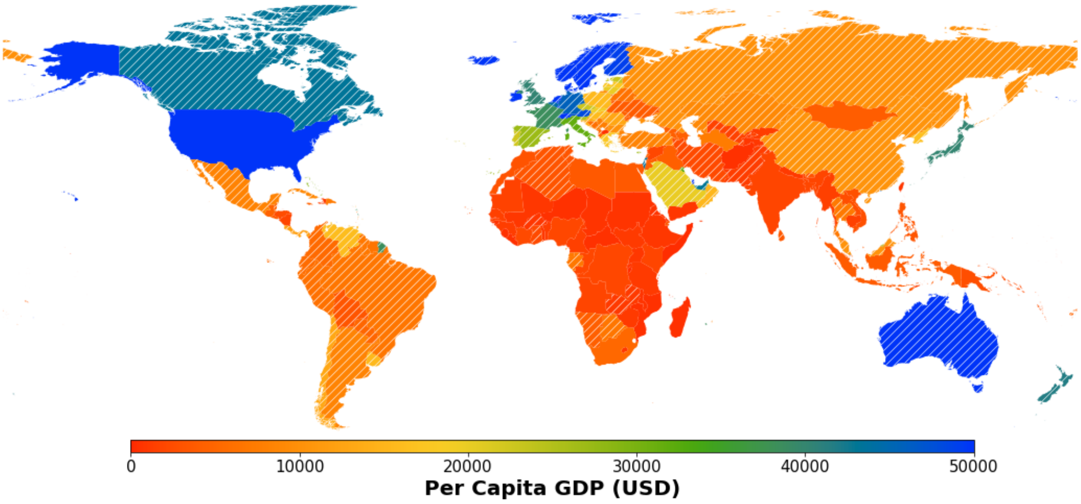

Figure S3. Country-specific per capita Gross Domestic Product, defined as the total value of goods and services produced in a country per person in USD every year. The hatched lines represent countries with universal healthcare. Countries without data are represented in gray.

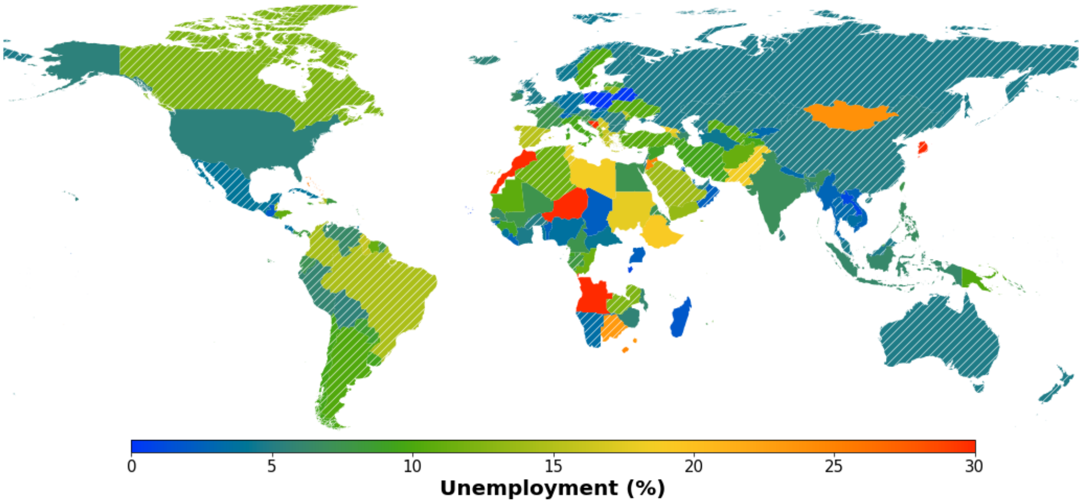

Figure S4. The country-specific proportion of individuals seeking employment in the labor force (people of working age). The hatched lines represent countries with universal healthcare. Countries without data are represented in gray.

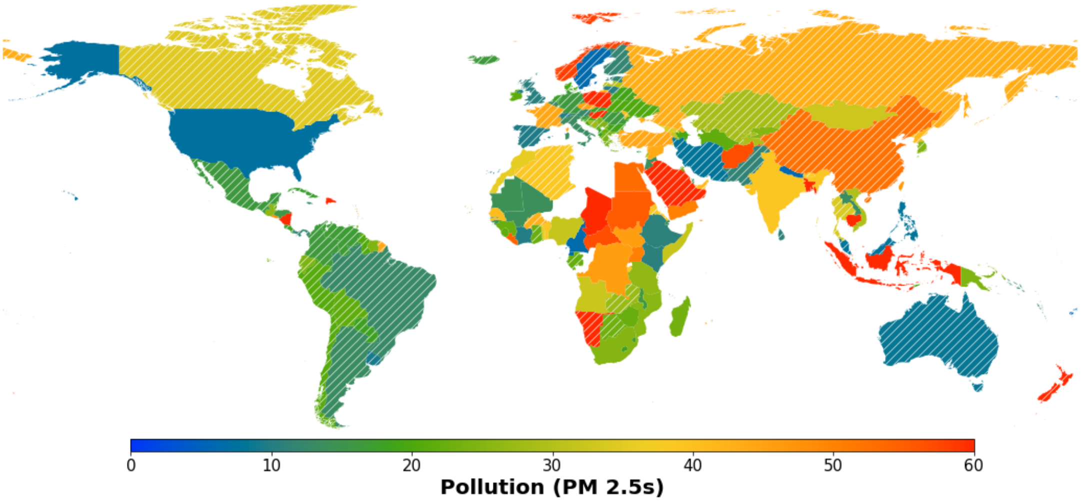

Figure S5. The mean level of national exposures to PM2.5: Population-weighted annual averages in μg/m3 units.The hatched lines represent countries with universal healthcare. Countries without data are represented in gray.

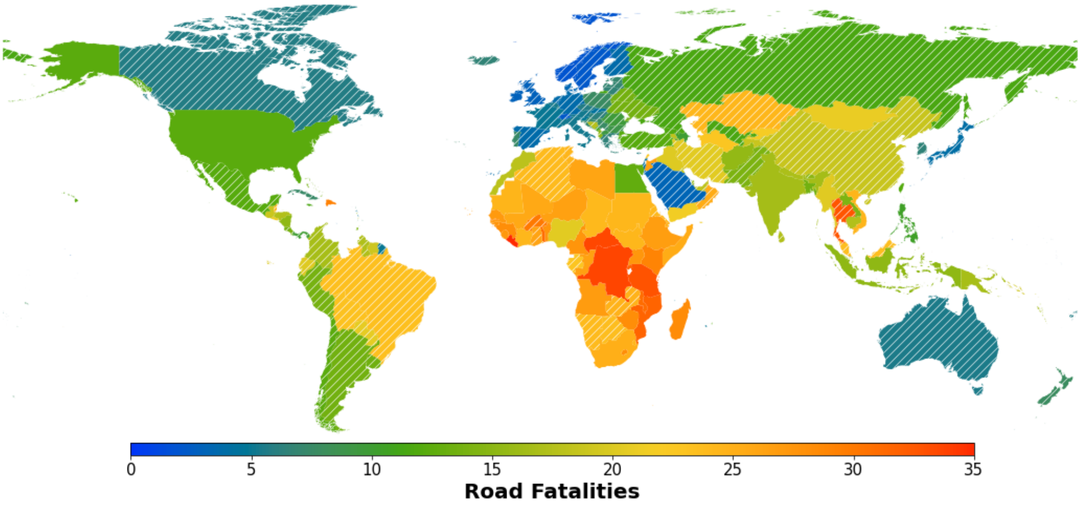

Figure S6. Country-specific number of road fatalities per 100,000 inhabitants per year. The hatched lines represent countries with universal healthcare. Countries without data are represented in gray.

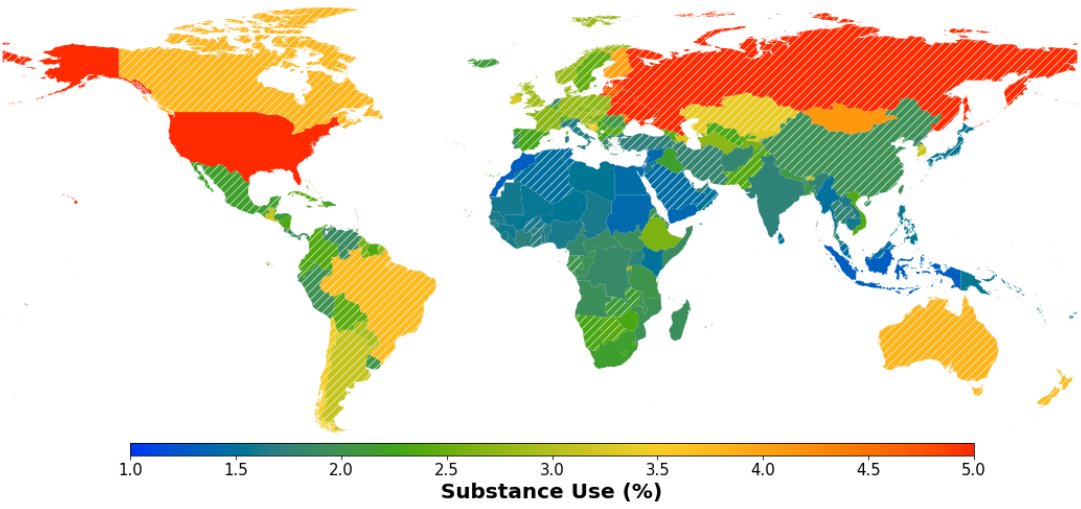

Figure S7. Country-specific proportion of population with substance use, which is defined as alcohol or drug use related disorders. The hatched lines represent countries with universal healthcare. Countries without data are represented in gray.

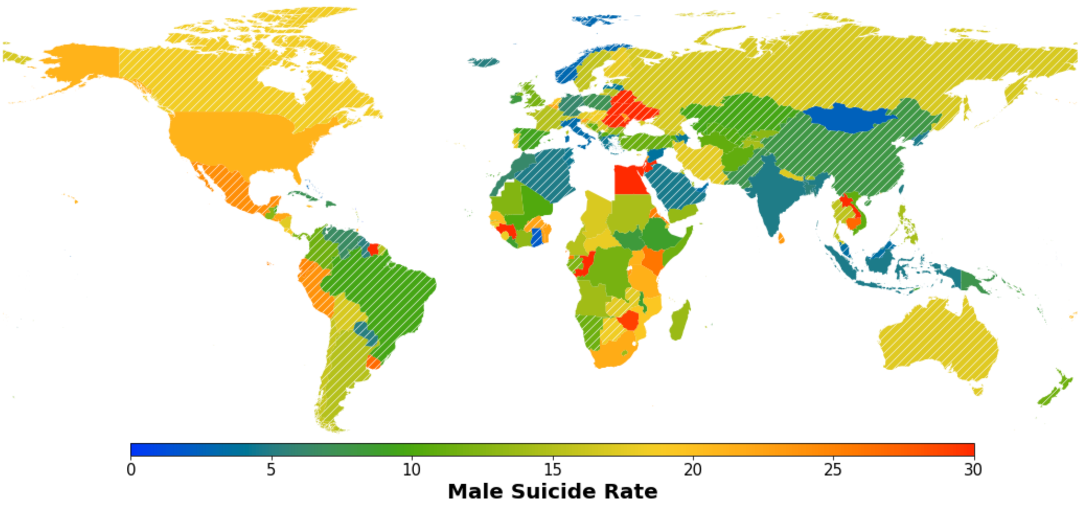

Figure S8. Country-specific number of male suicides per 100,000 male population. The hatched lines represent countries with universal healthcare. Countries without data are represented in gray.

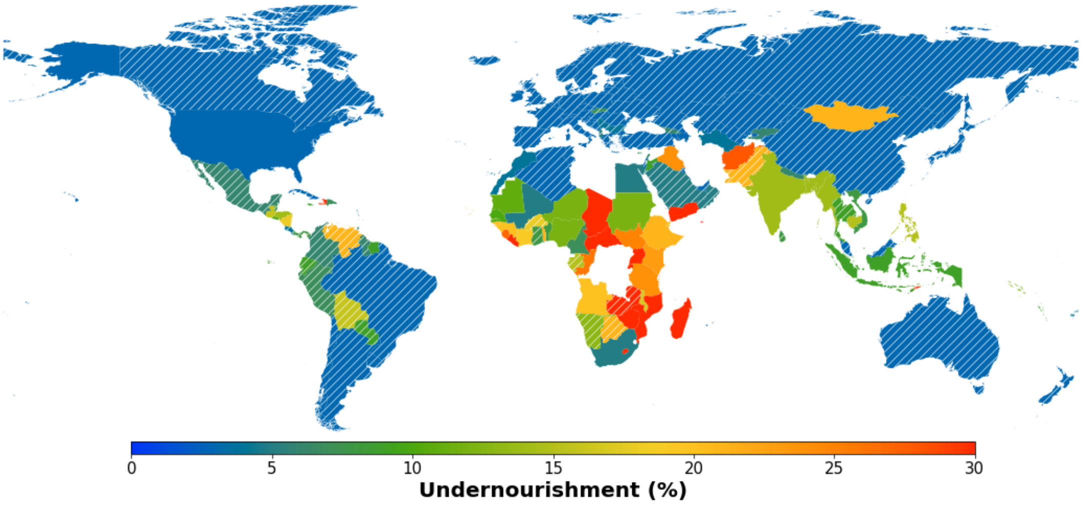

Figure S9. Country-specific prevalence of undernourishment, defined as……. The hatched lines represent countries with universal healthcare. Countries without data are represented in gray.

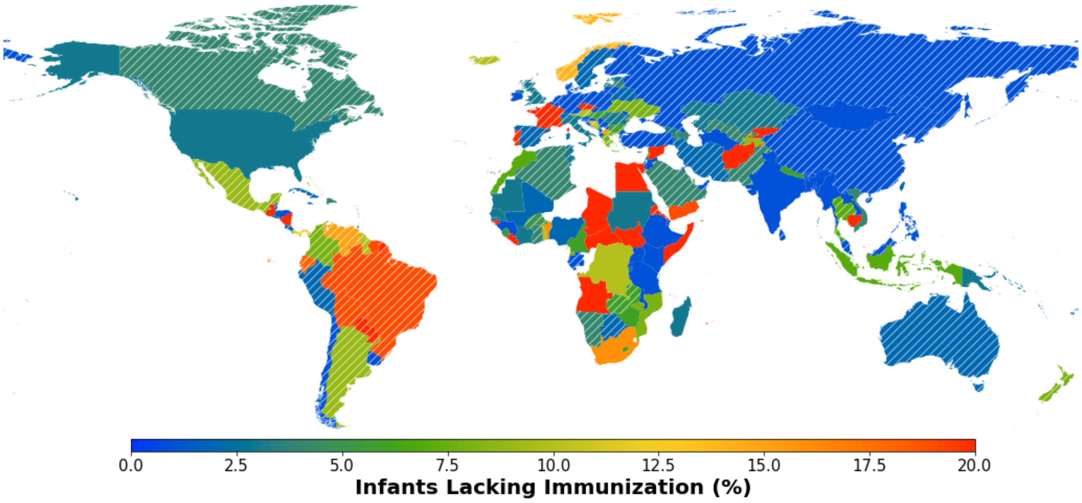

Figure S10. Country-specific percentage of one-year-olds who lack DTP immunization. The hatched lines represent countries with universal healthcare. Countries without data are represented in gray.

References

1. Literacy rate, adult total (% of people ages 15 and above). https://data.worldbank.org/indicator/SE.ADT.LITR.ZS.
2. Roser, M. & Ortiz-Ospina, E. Literacy. *Our World in Data* (2016).
3. Human Development Data (1990-2018). http://hdr.undp.org/en/data.
4. Educational attainment, at least completed lower secondary, population 25+, total (%) (cumulative). https://data.worldbank.org/indicator/SE.SEC.CUAT.LO.ZS?end=2019&start=2019&view=map.
5. GDP per capita (current US$). https://data.worldbank.org/indicator/NY.GDP.PCAP.CD.
6. Bank, W. Unemployment, total (% of total labor force)(modeled ILO estimate). (2017).
7. Wikipedia contributors. List of countries by unemployment rate. *Wikipedia, The Free Encyclopedia* https://en.wikipedia.org/w/index.php?title=List_of_countries_by_unemployment_rate&oldid=975771640 (2020).
8. Brauer, M. PM2. 5 air pollution, mean annual exposure (micrograms per cubic meter). *Retrieved from The World Bank: https://data. worldbank. org/indicator/EN. ATM. PM25. MC. M* **3**, (2016).
9. Data & Resources. https://www.stateofglobalair.org/engage.
10. Wikipedia contributors. List of countries by traffic-related death rate. *Wikipedia, The Free Encyclopedia* https://en.wikipedia.org/w/index.php?title=List_of_countries_by_traffic-related_death_rate&oldid=905091488 (2019).
11. Organization, W. H. & Others. GHO by category road traffic deaths data by country. (2013).
12. Ritchie, H. & Roser, M. Drug Use. *Our World in Data* (2019).
13. Human Development Data (1990-2018). http://hdr.undp.org/en/data.
14. Wikipedia contributors. List of countries by suicide rate. *Wikipedia, The Free Encyclopedia* https://en.wikipedia.org/w/index.php?title=List_of_countries_by_suicide_rate&oldid=974424835 (2020).

15. Prevalence of undernourishment (% of population) | Data. https://data.worldbank.org/indicator/sn.itk.defc.zs?end=2016&start=2000&view=chart.

16. Roser, M. & Ritchie, H. Hunger and undernourishment. *Our World in Data* (2013).

17. Human Development Data (1990-2018). http://hdr.undp.org/en/data.
